# Geospatial Assessment of Floods in Western Nepal

**DOI:** 10.1155/2021/8822846

**Published:** 2021-06-15

**Authors:** Sugam Subedi, Gandhiv Kafle, Shankar Tripathi

**Affiliations:** Faculty of Forestry, Agriculture and Forestry University, Makawanpur, Hetauda, Nepal

## Abstract

Floods are major problems, and their coexistence poses a potent threat, which cannot be eradicated but has to be managed. Extreme affects untold numbers of people, taxing economies, disrupting food production, creating unrest, and prompting migrations. There is much more that can be done to understand the effects of floods, particularly to help protect the poorest and most vulnerable. This research was carried out in the affected area of Bhimdatta municipality and aimed to find out the flood event of 2013 and present the scenario done for flood disaster management. The primary data were collected by direct observation and key informant survey. Landsat images were downloaded from USGS websites, and secondary information was collected through previous research and articles. The data were analyzed by using ArcGIS. It was found that the flood had created a negligible impact on the forest, high impact on the river itself, and average impact on land. 0.13% of forests, 17.38% of land, and 82.48% of river bodies were affected by the flood of 2013. Different governmental and nongovernmental organizations played an effective role for flood disaster management.

## 1. Introduction

Floods are the most common natural disasters that affect the societies around the world. In recent times, floods have gained increasing global significance as a result of their destructive nature and for causing monetary and human losses [1]. The effectiveness of floods is a function of several criteria such as flood power, magnitude, frequency, duration of the flow, changes of the planform, and cross-section geometry in a river [2]. Flooding is new to the Terai district of Nepal. The rivers in Terai are covered with silt and eroded through the Siwalik Range, which raises the bed level of the river and increases the risk of floods [3]. It is estimated that more than one-third of the world land area is flood prone, affecting nearly 82% of the world population [4]. Of all the disasters reported in Nepal, floods are the most devastating in terms of the number of deaths that occur and the damage they cause. A study by the UNDP ranked Nepal as the 30th country with respect to relative vulnerability to flood [5]. Nepal suffers a death of 300 people annually on an average only through floods [6]. Understanding the spatial change by flood and knowing disaster management practices will contribute to preparation of long-term disaster management plan.

More than 50 small streams have emerged in Chure of Kanchanpur district due to widespread deforestation carried out during the haphazard construction of roads making indirect cause to floods [7]. Careful monitoring as well as early warning for the flood is a big challenge for its management in Nepal [6]. Several government and nongovernment agencies have done research regarding loss [8] and hydrological analysis, but understanding through land cover analysis did not become a priority. Remote sensing data can potentially be used to estimate the extent and some degree of the severity of land cover changes or damages induced by floods [9]. Here, the remote sensor acquires a response which is based on many characteristics of the land surface, including natural or artificial cover [10]. It will also be helpful for the municipality to develop a land-use plan. Further understanding the overall postdisaster management at the local level till date will bring more insight about its implementation, style, and effectiveness.

Experience shows that the adverse impact of flood disasters can be reduced substantially if appropriate disaster preparedness plans and mitigation measures are developed and implemented [11], and our objectives support this reason. The objective of this study was to elucidate the land cover changes due to the latest major flood of 2013 in Bhimdatta municipality for accessing the area affected.

## 2. Materials and Methods

### 2.1. Study Area

**Figure 1:**
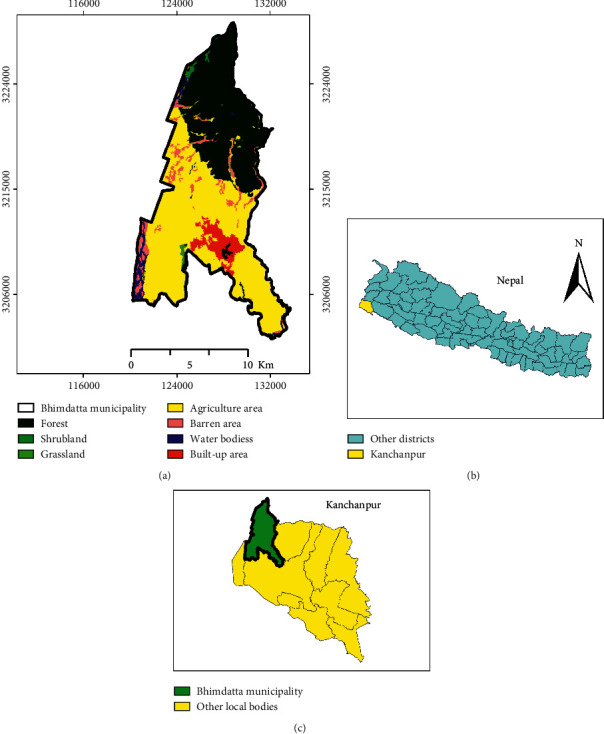
Map of the study area.

The study was carried out in the Bhimdatta municipality of Kanchanpur district in Nepal as shown in the map of the district (Figure 1). The major river creating flood here is Mahakali. Ward numbers 11, 12, and 13 were most affected by flood, and these wards were studied for ground and key informant survey. The population of Bhimdatta municipality was 104,444 according to the 2011 Central Bureau of Statistics Data. The precipitation on the Dipayal hydrological station was up to 210 mm during June 2013, and the river discharge was 550 cusec in the Mahakali Sharada Barrage station [12]. Land covered by water during regular flow was 432 ha, whereas forests covered 5796 ha, agriculture land covered 8913 ha, and barren land covered 1072 ha.

### 2.2. Data Collection

#### 2.2.1. Primary Data as Satellite Images

Landsat 8 OLI images were used due to their satisfaction in performance [10] (Table 1). They were downloaded from USGS GloVis (http://www.glovis.usgs.gov) for free of cost. The boundary of the study area was collected from the department of survey. Both images were stacked and clipped by the study area using ArcGIS. Topographic correction was not conducted as the study had no sloppy area and shadow place. Training samples for the year 2013 were collected from Google Earth Pro by using the previous scene function. The maximum likelihood classifier of the supervised classification technique has been used in which a pixel with the maximum likelihood is classified into the corresponding class [13].

#### 2.2.2. Abstraction of the Affected Area

The maps of pre- and postflood are vectorized after image classification. After the vectorization process, clipping of water bodies is done on the postflood map which is the collection of preland, forest, and water itself and gives us only affected cover through it by using ArcGIS.

#### 2.2.3. Key Informant Survey

Key informants such as ward chairperson, environmental officer administrative officer from the municipality, police officers involved in early warning disaster management, and NGO head and other different stakeholders were interviewed regarding the flood issues.

## 3. Results and Discussion

### 3.1. Land Cover Maps

**Figure 2:**
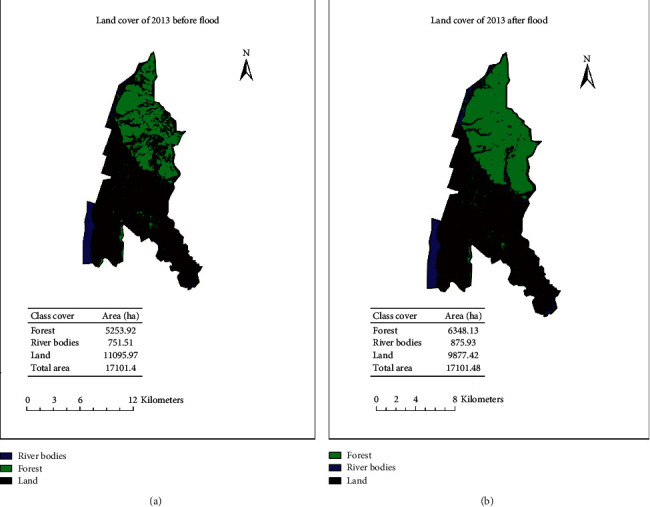
Land cover image before and after flood.

Figure 2 represents how land cover changed during the two months, but it is not due to the flood but due to the overall changes. Das [1] indicated that the output flood risk map can be useful for planners, managers, and regulatory bodies to manage and mitigate flood incidents.

**Figure 3:**
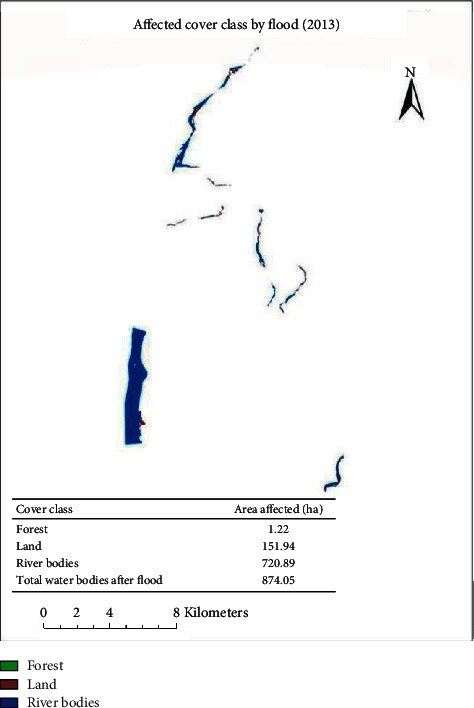
Affected cover class by flood (2013).

The forest area is changed by 1094.21, river bodies by 124.42, and land area by 1218.55 ha (Figure 3). Das [14] prepared a resultant map which showed that about 20% of the total area in the Vaitarna basin is having a very high probability of flood, and these regions require some serious attention of governmental or nongovernmental bodies to reduce the flood risk.

### 3.2. Affected Land Cover

The actual land cover which was changed to total water bodies by flood is 874.05 ha: from forest (1.22 ha), from land (151.94 ha), and from previous river bodies (720.89 ha) (Figure 3). The flood susceptibility map is constructed based on twelve influencing parameters, i.e., elevation, slope, distance from the drainage network, geomorphology, drainage density, flow accumulation, rainfall, land use, geology, stream power index, topographic wetness index, and curvature of the topography [2].

### 3.3. Disaster Management Scenario

#### 3.3.1. Status of the Community-Based Flood Early Warning System

  Existence of a specific committee of people concerned on the flood early warning system village wise in each affected ward.
  Coordination of the committee with District Emergency Disaster Management Office and communication during emergencies.
  Use of equipment such as the whistle, mobile phone, siren, and audio devices to make people alert especially during nighttime and emergencies.
  Four members from each committee get officially trained.

#### 3.3.2. Flood and Watershed Management Practices (Present Scenario)

Increases in river bodies' area followed by land due to flood of 2013 was immense. It showed that major focus must be given to control the riverside area including settlements and agricultural land rather than forests, and accordingly, it was found that work has been done for those areas. The research also showed that a good coordination between stakeholders and implementation of disaster management programs through different organizations actually bring positive changes for being prepared and reducing the impact (Table 2). Das [14] mentioned that flood damage can be reduced through implementing proper management and policies.

The recent laws formulated on disaster management are as follows:

- MoHA has made Monsoon Emergency Operation Plan, 2076, for risk management, reduction, rescue, and relief distribution
- Formulation of Disaster Risk Reduction and Management Regulation, 2076
- Formulation of Integrated Settlement Development Regulation, 2075
- Management of Dead Body after Disaster Guideline, 2068, First Amendment, 2076

The result of the study revealed that flood might affect increasing river area with the change of its course, and for this, it must be managed which is done by the construction of a big six-lane Mahakali corridor which maintains the future flood to be in track.

CBFEWS must be run with the help of updated technical reforms which is a greater part in lowering impacts. There is conflict of power between Soil and Watershed conservation office Dadeldhura and Watershed Management committee Mahakali, Baitadi as their Mahakali Basin Management comes under both offices.

## 4. Conclusions

Land cover change due to flood has created more effect to land area externally and a negligible impact on forests of Bhimdatta municipality. Different procedures such as developing gravel mining guidelines, the use of communication media, infrastructural development, especially 6-lane roads, different awareness, and educative programs have contributed for watershed and flood disaster management. Participatory flood management with the application of technical principles seems effective in reducing effects of the floods. Furthermore, solving issues of people regarding their flooded lands through compensations and developing rules on riverside settlement are must in such mega watershed areas. The research work gives a picture for prioritizing zones for flood disaster management and also signifies what problems do exist in such mega watershed areas that need to be solved in the long run.

## Figures and Tables

**Table 1:** Landsat images used.

Landsat images	Spatial resolution	Date acquired	Cloud cover (%)
Landsat 8 OLI	30	2013/06/13	2
Landsat 8 OLI	30	2013/08/03	7

**Table 2:** Present scenario of flood and watershed management.

Organizations and projects	Works done
USAID Paani project	(i) Watershed protection like source conservation program
	(ii) Conservation education on aquatic biodiversity is provided

NEEDS Kanchanpur	(i) Preparation of gravel mining guidelines which helps for its basin management by appropriate utilization of river resources
	(ii) Hazard map preparation in respective villages
	(iii) Radio communications, road play, advocacy, and meeting with different stakeholders are being done

Bhimdatta municipality	(i) Preparation of disaster risk reduction and management regulation, 2075, by Bhimdatta municipality has been supported for flood disaster management
	(ii) Allocation of annual budget ward wise for disaster risk management

Government of Nepal	(i) Establishment of “Mega Watershed Management Committee, Mahakali,” at Baitadi as the new office under the central government for larger basin management
	(ii) Six-lane road construction which acts as a dam for the protection of villages from the river is being completed
	(iii) Another remarkable work is the construction of a four-lane bridge of 800 m joining Mahakali and Bhimdatta municipality by narrowing the width which was 2300 m of Mahakali in the construction site by Nepal Government itself

## Data Availability

The data used to support the findings of this study may be released uponapplication to the first author, who can be contacted at gkafle@afu.edu.np.
